# Promising inhibitors targeting M^pro^: an ideal strategy for anti-SARS-CoV-2 drug discovery

**DOI:** 10.1038/s41392-020-00291-8

**Published:** 2020-08-27

**Authors:** Yi Chen, Guan Wang, Liang Ouyang

**Affiliations:** State Key Laboratory of Biotherapy and Cancer Center & Department of Gastrointestinal Surgery, West China Hospital, and Collaborative Innovation Center of Biotherapy, Sichuan University, 610041 Chengdu, China

**Keywords:** Infectious diseases, Medicinal chemistry

**Fig. 1:**
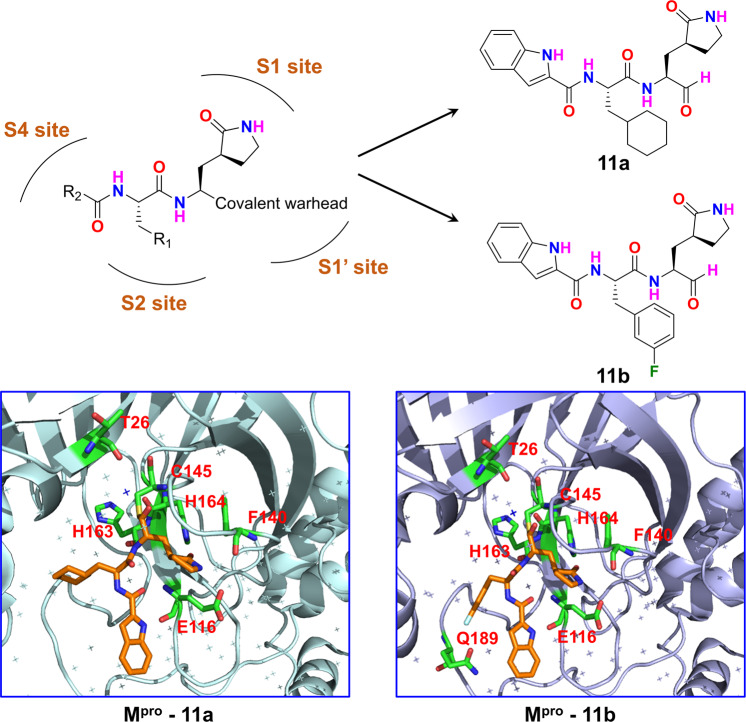
Design and discovery of drug candidates targeting M^pro^ against COVID-19

Recently, Dai W et al. published a study on Science,^1^ in which the two lead compounds **11a** and **11b** were designed and synthesized based on the features of a key enzyme M^pro^ of SARS-CoV-2 (Fig. 1). In particular, compound **11a** is a potential drug candidate for coronavirus disease 2019 (COVID-19) with strong anti-SARS-CoV-2 infection activity, good pharmacokinetics characteristics, and low toxicity.

Since December 2019, the outbreak of COVID-19 caused by SARS-CoV-2^2^ has caused a serious global public health emergency. So far, the global epidemic is still in the outbreak stage, and the number of new confirmed cases every day has exceeded 100,000 for several days. At present, the main drugs used clinically include interferon-alpha, lopinavir/ritonavir, ribavirin, arbidol, etc. However, these drugs are facing huge controversy due to the large side effects or the lack of clinical verifications of the therapeutic effects.^3^ Therefore, clarifying the origin and pathogenesis of pneumonia and decoding the key targets against SARS-CoV-2 are the cornerstone to design and discover safe and effective antivirus drugs.

Previously, the crystal structure of the M^pro^ protein of SARS-CoV-2 was resolved in complex with an effective inhibitor **N3**, which was completed by the same group, laying an important foundation for this research.^4^ M^pro^ plays an irreplaceable role in the life cycle of the virus in the light of it could release a series of functional peptides by hydrolyzing the two proteins necessary for replication and transcription, pp1a and pp1ab.^5^ Notably, conservatism in coronavirus and lack of homolog in human also enhance the application of M^pro^ in antiviral drug design.^5^

The active sites of M^pro^, which usually include S1’, S1, S2, and S4, are highly conserved in all coronaviruses. Accordingly, the rational design can be applied in the discovery of novel SARS-COV-2 inhibitors. Because SARS-CoV M^pro^ inhibitors usually have (S)-γ-lactam ring to occupy the S1 site, (S)-γ-lactam ring is introduced to interact with the S1 site. Furthermore, an aldehyde group is selected to form a covalent bond with the thiol of the Cys145 residue. The S2 region is capable to accommodate a large group, so a cyclohexyl or 3-fluorophenyl group with large spatial volume is introduced at the corresponding position. Afterwards, an indole group is placed in the S4 region to form intermolecular hydrogen bonds so as to improve drug-like properties. Finally, a synthetic route is developed to afford the lead compounds **11a** and **11b**.

Next, activity, pharmacokinetics properties and toxicity of **11a** and **11b** were evaluated. In a fluorescence resonance energy transfer (FRET)-based cleavage assay, both compounds **11a** and **11b** exhibited strong inhibitory activities, with the potency of IC_50_ = 0.053 ± 0.005 μM and 0.040 ± 0.002 μM, respectively. A further assay revealed that **11a** and **11b** also displayed good antiviral activities in Vero E6 cells (EC50 of 0.53 ± 0.01 μM and 0.72 ± 0.09 μM, respectively) with low cytotoxicity (CC50 is greater than 100 μM). Finally, pharmacokinetics and toxicity studies showed that **11a** had better pharmacokinetics properties and no obvious toxicity in vivo.

To elucidate the inhibitory mechanism of **11a** and **11b**, the crystal structure of M^pro^ in complex with **11a** (PDB code: 6LZE) and **11b** (PDB code: 6M0K) was resolved, respectively, at a resolution of 1.5 Å. The structures show that **11a** and **11b** have a similar inhibitory binding mode. The aldehyde group forms a covalent force with Cys145 in the S1’ region, whereas (S)-γ-lactam ring and indole group form intermolecular hydrogen bonds with S1 and S4 regions, respectively. A subtle difference between **11a** and **11b** in the S2 site was most probably due to the stereostructure and electronegativity difference between cyclohexyl and 3-fluorophenyl groups. Notably, multiple water molecules also participated in the binding of protein—ligand complexes via hydrogen bonds. Overall, the binding modes of **11a** and **11b** with the M^pro^ are consistent with those of compounds **N1**, **N3**, and **N9** that are reported as wide spectrum inhibitors targeting coronavirus M^pro^.

Taken together, with no vaccine or proven effective drug against SARS-COV-2, Dai W et al. designed and synthesized effective inhibitors based on the structure of the specific target M^pro^ active site. Compound **11a** is expected to become a promising clinical drug candidate. This research provides an effective strategy to design and discover anti-SARS-CoV-2 and even anti-coronavirus drugs targeting M^pro^. Before the emergence of specific drugs, the comprehensive application of drug design, medicinal chemistry, multidisciplinary technologies such as structural biology will help accelerate the development of anti-COVID-19 drugs.
